# Severe Acute Respiratory Syndrome Coronavirus-2 Inactivation Activity of the Polyphenol-Rich Tea Leaf Extract with Concentrated Theaflavins and Other Virucidal Catechins

**DOI:** 10.3390/molecules26164803

**Published:** 2021-08-08

**Authors:** Yohei Takeda, Kyohei Tamura, Dulamjav Jamsransuren, Sachiko Matsuda, Haruko Ogawa

**Affiliations:** 1 Research Center for Global Agromedicine, Obihiro University of Agriculture and Veterinary Medicine, 2-11 Inada, Obihiro 080-8555, Japan; ytakeda@obihiro.ac.jp; 2 Department of Veterinary Medicine, Obihiro University of Agriculture and Veterinary Medicine, 2-11 Inada, Obihiro 080-8555, Japan; s16110021@st.obihiro.ac.jp (K.T.); duuya.dj@gmail.com (D.J.); chaka@obihiro.ac.jp (S.M.)

**Keywords:** catechin, polyphenol, SARS-CoV-2, tea leaf extract, theaflavin, virucidal activity

## Abstract

Since severe acute respiratory syndrome coronavirus 2 (SARS-CoV-2) is producing a large number of infections and deaths globally, the development of supportive and auxiliary treatments is attracting increasing attention. Here, we evaluated SARS-CoV-2-inactivation activity of the polyphenol-rich tea leaf extract TY-1 containing concentrated theaflavins and other virucidal catechins. The TY-1 was mixed with SARS-CoV-2 solution, and its virucidal activity was evaluated. To evaluate the inhibition activity of TY-1 in SARS-CoV-2 infection, TY-1 was co-added with SARS-CoV-2 into cell culture media. After 1 h of incubation, the cell culture medium was replaced, and the cells were further incubated in the absence of TY-1. The viral titers were then evaluated. To evaluate the impacts of TY-1 on viral proteins and genome, TY-1-treated SARS-CoV-2 structural proteins and viral RNA were analyzed using western blotting and real-time RT-PCR, respectively. TY-1 showed time- and concentration-dependent virucidal activity. TY-1 inhibited SARS-CoV-2 infection of cells. The results of western blotting and real-time RT-PCR suggested that TY-1 induced structural change in the S2 subunit of the S protein and viral genome destruction, respectively. Our findings provided basic insights in vitro into the possible value of TY-1 as a virucidal agent, which could enhance the current SARS-CoV-2 control measures.

## 1. Introduction

Severe acute respiratory syndrome coronavirus 2 (SARS-CoV-2) is an enveloped single-stranded RNA virus belonging to the genus *Betacoronavirus* and was first identified in December of 2019 in China [1,2]. As the causative agent of the coronavirus disease 2019 (COVID-19) pandemic, it is still causing a very large number of infections and deaths worldwide [3]. Restricting the spread of pathogenic viruses, reducing the number of infections and deaths, and eventually ending the pandemic are clearly important. There are three main types of virus control measures: (1) prevention of respiratory and contact transmissions by reducing the chance of virus inhalation and removing viruses from environmental surfaces and hands using disinfectants and hand washing, (2) treatment of infections through the use of medication, and (3) induction of herd immunity by vaccination.

Many disinfectants that can efficiently inactivate SARS-CoV-2 on surfaces in a short time have been reported [4,5,6]. However, the main transmission mode of SARS-CoV-2 is respiratory infection via droplet inhalation, and contact transmission is considered to be rare [7]; therefore, the effect of inactivating viruses on environmental surfaces and hands might be limited.

With respect to point 2, many types of approved or experimental drugs have been investigated for COVID-19 treatment. The main types of drugs are RNA-dependent RNA polymerase blockers such as remdesivir; neutralizing monoclonal antibodies; and corticosteroids such as dexamethasone, Janus kinase inhibitor, and anti-IL-6 receptor antibody, which inhibit excessive inflammation induced by SARS-CoV-2 infection [8,9]. However, the therapeutic efficacies of these drugs are not absolute and are not effective in all COVID-19 cases.

Vaccines are the third line of defense against viruses, and several different vaccines have been approved for use in different countries. However, vaccination status differs among countries, and there remain many countries in which widespread vaccination will not be achieved for several months or more. Some variant strains, especially the B.1.351 lineage variant (Beta strain), which was first reported in South Africa, have shown a capacity to escape from the neutralizing antibodies established using the original strain [10,11]. These findings indicate the concern that herd immunization against novel variant strains which will appear in the future may not be achievable using currently available vaccines. The current SARS-CoV-2 control measures are therefore still insufficient to bring the pandemic under control, and the development of additional supportive and auxiliary approaches, especially those that are easily implemented by the general public, are required.

The application of naturally derived, especially plant-based, compounds for supportive and auxiliary treatments is attracting increasing attention. Phytochemicals have a wide variety of biological activities, including broad antimicrobial activities. Medicinal plant-derived compounds tend to be low toxic, and more than 50% of medical drugs used in Western countries are derived from plant-based compounds. Several antiviral phytochemicals have been identified, and the potential application of these compounds to the treatment of viral diseases has been suggested [12]. Phenolics, carotenoids, terpenoids, and alkaloids are well-known antiviral phytochemicals. In the phenolics family, phenolic acids, flavonoids, stilbenes, coumarins, and tannins are known to have antiviral activities [13,14]. The virucidal and antiviral activities of naturally derived polyphenols are widely known; they have been reported to prevent viral absorption and entry to host cells and inhibit viral genome and protein synthesis in infected cells [14]. Catechin is one of the flavonoids contained in the leaves of green tea (*Camellia* *sinensis*). Green tea catechins, including catechin, epicatechin (EC), epicatechin gallate (ECG), epigallocatechin (EGC), and epigallocatechin gallate (EGCG), have antitumor, antioxidative, antibacterial, and antiviral activities against multiple pathogenic virus species [15]. Fermented black tea contains theaflavins (TFs), which are dimers of catechins with a benzophenone structure. Although TFs are considered to show more potent biological activities than green tea catechins and the quality of black tea partially depends on TF content, TF content is low in many black teas [16,17]. The chemical structures of TFs and green tea catechins are described in an article by Liu et al. [18]. Since TFs, as well as green tea catechins, have been reported to show antiviral activity against various viral species [19], these antiviral catechin-rich components may be valuable as SARS-CoV-2 control measures. In this study, we evaluated the SARS-CoV-2 inactivation activity of a polyphenol-rich tea leaf extract, named TY-1, containing green tea catechins and concentrated TFs [20], and discuss its possible usage for the prevention or treatment of COVID-19.

## 2. Results

### 2.1. Virucidal Activity of TY-1 against SARS-CoV-2

**Figure 1:**
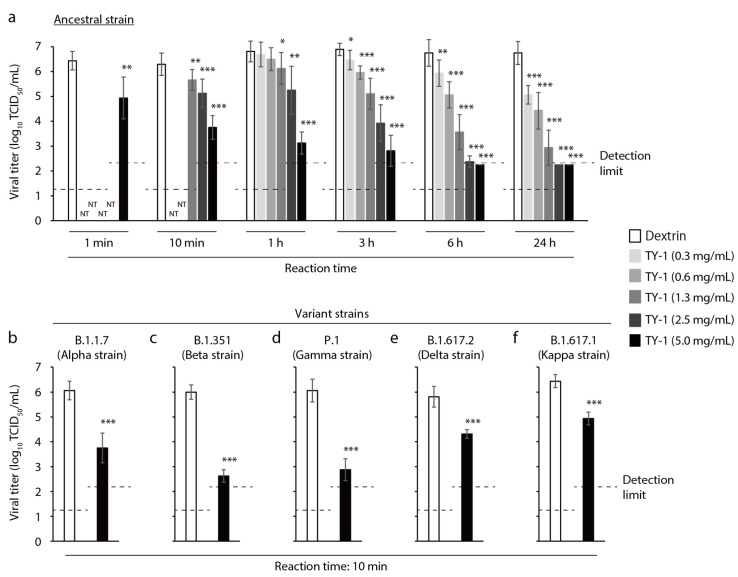
Virucidal activity of TY-1 against SARS-CoV-2. (**a**)–(**f**) SARS-CoV-2 solution ((**a**) ancestral strain, (**b**) Alpha strain, (**c**) Beta strain, (**d**) Gamma strain, (**e**) Delta strain, (**f**) Kappa strain) was mixed with TY-1 at several different concentrations. As a diluent control, dextrin (2.5 mg/mL) was mixed with the viral solution. The mixtures were incubated at 22–25 °C from 1 min to 24 h (**a**) or for 10 min (**b**)–(**f**); then, the viral titers were evaluated. The detection limits of the viral titer were 1.25 log_10_ TCID_50_/mL in the dextrin and TY-1 (0.3–1.3 mg/mL) groups and 2.25 log_10_ TCID_50_/mL in the TY-1 (2.5 and 5.0 mg/mL) groups. The results are indicated as mean ± SD (*n* = 4–12 per group). Student’s *t*-tests were performed to evaluate the statistical significance of the differences between the dextrin and each TY-1 groups; * *p* < 0.05; ** *p* < 0.01; *** *p* < 0.001; NT: not tested.

The virucidal activities of solutions with different concentrations of TY-1 against the ancestral strain and multiple variant strains of SARS-CoV-2 were evaluated. TY-1 solution showed time- and concentration-dependent virucidal activity against the ancestral strain. At 3-h to 24-h reaction times, the viral titers in all tested concentrations of TY-1 were significantly lower than those of the dextrin group. At 6-h and 24-h reaction times, the viral titers in the 2.5 and 5.0 mg/mL TY-1 groups were almost below the detection limit (2.5 mg/mL TY-1: ≥ 4.38 log_10_ 50% tissue culture infective dose [TCID_50_]/mL reduction; 5.0 mg/mL: ≥ 4.50 log_10_ TCID_50_/mL reduction compared with the dextrin group at 6 h). At 10-min reaction time, the significant reductions of viral titers in the 1.3, 2.5, and 5.0 mg/mL TY-1 groups were 0.63, 1.17, and 2.54 log_10_ TCID_50_/mL, respectively. The 5.0 mg/mL TY-1 group also showed statistically significant virucidal activity at 1-min reaction time, and the reduction of the viral titer was 1.5 log_10_ TCID_50_/mL (Figure 1a). The significant reductions of viral titers in the 5.0 mg/mL TY-1 group were 2.31, ≥ 3.34, ≥ 3.19, 1.50, and 1.50 TCID_50_/mL against Alpha, Beta, Gamma, Delta, and Kappa strains at 10-min reaction time, respectively (Figure 1b–f).

### 2.2. Inhibitory Effects of TY-1 on SARS-CoV-2 Infection of Cells

**Figure 2:**
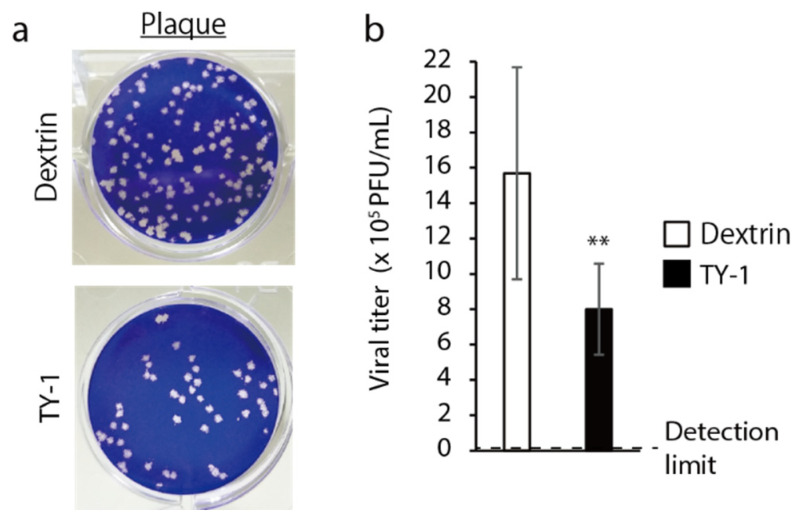
Inhibitory effect of TY-1 on SARS-CoV-2 infection of cells. (**a**,**b**): SARS-CoV-2 (ancestral strain)-added cells were incubated at 37 °C for 1 h in the presence of dextrin (25 μg/mL) or TY-1 (50 μg/mL); then, the cell culture media was removed. The cells were additionally incubated in 1.55% carboxymethyl cellulose sodium salt-containing virus growth medium (VGM) without dextrin or TY-1 at 37 °C for 3 d. (**a**) Plaques in representative wells, inoculated with 10^4^-times diluted SARS-CoV-2 are shown. (**b**) The viral titer (× 10^5^ PFU/mL) in each test group is shown. The detection limit of the viral titer was 0.00004 × 10^5^ PFU/mL. The results are indicated as mean ± SD (*n* = 8 per group). Student’s *t*-tests were performed to evaluate the statistical significance of the differences between the dextrin and each of the TY-1 groups; ** *p* < 0.01.

SARS-CoV-2-added cells were incubated for 1 h in the presence of 25 μg/mL dextrin or 50 μg/mL TY-1, and inhibition by TY-1 of the viral infection in the cells was evaluated. The number of plaques was fewer in the TY-1 group than in the dextrin group (Figure 2a). The viral titer was significantly lower in the TY-1 group than in the dextrin group. The viral titer in the dextrin group was 15.68 × 10^5^ plaque-forming unit (PFU)/mL and that in the TY-1 group was 8.00 × 10^5^ PFU/mL (Figure 2b).

### 2.3. Impact of TY-1 on SARS-CoV-2 Structural Proteins

**Figure 3:**
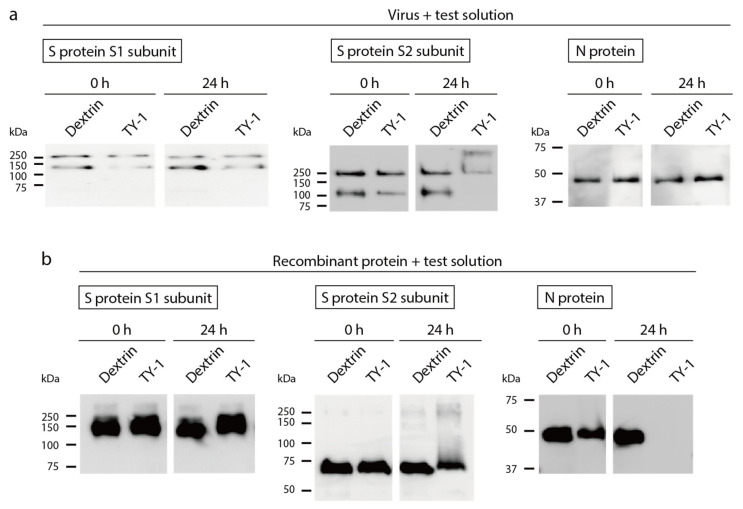
Impact of TY-1 on SARS-CoV-2 structural proteins. (**a**) SARS-CoV-2 (ancestral strain) was mixed with dextrin (2.5 mg/mL) or TY-1 (5.0 mg/mL). (**b**) The recombinant S1 subunit of the S protein, the S2 subunit of the S protein, or the N protein was mixed with dextrin or TY-1. (**a**,**b**) After 0-h and 24-h reaction time, respectively, western blotting was performed. The pictures on the left, middle, and right show the results of western blotting to detect the S1 subunit, S2 subunit, and N protein, respectively.

SARS-CoV-2 solution was mixed with dextrin or TY-1 solutions, and the impact on viral structural proteins expressed in viral particles was evaluated using western blotting. There were no differences between the band patterns of the S1 subunit of the S protein, the S2 subunit of the S protein, or the N protein in the dextrin and TY-1 groups at 0-h reaction time. There were also no differences in the band patterns of the S1 subunit and the N protein between these two groups at 24-h reaction time (Figure 3a, left and right). The two S2 subunit-specific bands observed in the dextrin group disappeared or were weakened in the TY-1 group at 24-h reaction time. An additional band, with a higher molecular mass, appeared in the TY-1 group (Figure 3a, middle). To more specifically evaluate the impact of TY-1 on each structural protein, each recombinant protein was mixed with dextrin or TY-1 solution. Western blotting identified no differences between the dextrin and TY-1 groups at 0-h reaction time in the band patterns of the three proteins tested. There was also no difference in the band patterns of the S1 subunit between these two groups at 24-h reaction time (Figure 3b, left). The band intensity of the S2 subunit tended to be weakened, and an additional ladder with higher molecular mass appeared in the TY-1 group at 24-h reaction time (Figure 3b, middle). The N protein-specific band disappeared in the TY-1 group at 24-h reaction time (Figure 3b, right).

### 2.4. Impact of TY-1 on the SARS-CoV-2 Genome

**Figure 4:**
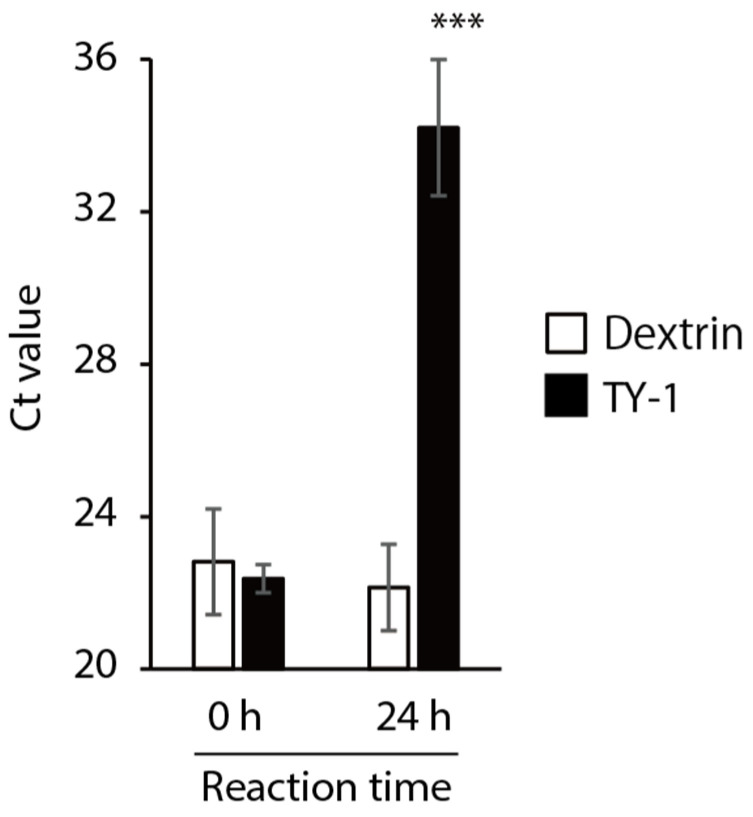
Impact of TY-1 on the SARS-CoV-2 genome. SARS-CoV-2 (ancestral strain) was mixed with dextrin (2.5 mg/mL) or TY-1 (5.0 mg/mL). After 0-h and 24-h reaction time, respectively, real-time RT-PCR was performed, and the Ct value was evaluated. The results are indicated as mean ± SD (*n* = 4 per group). Student’s *t*-tests were performed to evaluate the statistical significance of differences between the dextrin and TY-1 groups; *** *p* < 0.001.

SARS-CoV-2 solution was mixed with dextrin or TY-1 solution, and the impact on the viral genome was evaluated using real-time reverse transcription polymerase chain reaction (RT-PCR) analysis. There was no difference in the cycle threshold (Ct) value between the dextrin and TY-1 groups at 0-h reaction time. The Ct value was higher in the TY-1 group than in the dextrin group at 24-h reaction time. The difference in Ct value between these two groups was 12.06 (Figure 4). This result suggests that TY-1 induced the destruction of the viral genome.

## 3. Discussion

In this study, we found a SARS-CoV-2 inactivation activity of TY-1, which contains abundant polyphenols, including green tea catechins and concentrated TFs. TY-1 also contains 1.8% caffeine and 1.3% theanine (Table S1, in Supplementary Materials). Although there have been several reports that caffeine has some inhibitory activity against virus proliferation in infected cells, it did not appear to have clear virucidal activity [21]. Although it has been suggested that theanine contributes to enhanced antiviral immunity [22], there are almost no reports related to its virucidal activity. A direct impact of TFs has been reported against the viral particles of both enveloped and nonenveloped virus species, including influenza virus, human immunodeficiency virus (HIV), rotavirus, enterovirus, calicivirus, and hepatitis C virus [18,23,24,25,26]. The virucidal activities of green tea catechins against SARS-CoV-2, influenza A virus, calicivirus, and many other virus species have been also shown in previous studies by ourselves and other researchers [19,27]. Hence, the SARS-CoV-2 inactivation activity of TY-1 may largely depend on virucidal tea polyphenols such as TFs and other catechins. Some reports have suggested that several plant-derived polyphenols have additive and synergistic antiviral activities [28,29]. Multiple polyphenols could have contributed to the total SARS-CoV-2 inactivation activity of TY-1.

In this study, we showed that TY-1 treatment induced structural changes in the S protein of SARS-CoV-2 (Figure 3). Polyphenols, including catechins, bind to proteins [15,30], and previous reports have shown that TFs impact HA and NA proteins, the spike proteins of the influenza A virus, resulting in the inhibition of their functions [23,31]. In this study, a high-molecular-mass band/ladder appeared in the western blots of the TY-1-treated S2 subunit of the S protein. In our previous study, in which the SARS-CoV-2-inactivating activity of olive polyphenol-rich extract was demonstrated, the appearance of similar high-molecular-mass bands/ladders was observed for both the S1 and S2 subunits of the S protein [32]. These results indicate that some types of plant-based polyphenols may induce the aggregation of S proteins. While olive polyphenols impacted both the S1 and S2 subunits, TY-1 seemed to impact only the S2 subunit. The S1 subunit is responsible for the binding of SARS-CoV-2 to the viral receptor ACE2 on host cells, and the S2 subunit is responsible for the fusion of the virus envelope and host cell membrane [33]. Liu et al. [18] showed that TFs and EGCG did not clearly block HIV Env glycoprotein gp120 subunit-CD4 interaction but blocked the six-helix bundle formation in the gp41 subunit, resulting in the inhibition of virus envelope-cell membrane fusion. In the present study, a similar mechanism of action of the catechins in TY-1 on the S2 subunit may have contributed to the inhibition of SARS-CoV-2 infection of cells (Figure 2). Susceptibility to TY-1 seemed to be slightly different in the different SARS-CoV-2 strains. The susceptibility to TY-1 was highest in the Beta and Gamma strains, followed by the ancestral and Alpha strains. The susceptibility of the Delta and Kappa strains tended to be lower (Figure 1). Since there are multiple discrepancies in the amino acid sequences of the S protein among these different strains, such amino acid substitutions, especially in the S2 subunit, may impact the sensitivity to TY-1. The S2 subunit contains the fusion peptide, heptapeptide repeat 1 and 2 (HR1, HR2), the transmembrane domain, and the cytoplasm domain [34]. In the strains tested here, there were several discrepancies in the amino acid sequence in the HR1 and HR2 regions, but not in the fusion peptide or the transmembrane/cytoplasm domains. As HR1 and HR2 are indispensable to virus envelope-cell membrane fusion and virus entry, these regions may be a possible target of the virucidal compounds contained in TY-1. Future computational docking analysis of the S2 subunit and TFs/green tea catechins may contribute to further understanding of the details of their inactivation of SARS-CoV-2.

TY-1 also impacted uncovered recombinant N protein, but not N protein in viral particles. These differing results suggest that TY-1 interacts with the uncovered N protein, but the amount of TY-1 reaching the N protein located inside viral particles was possibly not enough to have an effect.

TY-1 also impacted the SARS-CoV-2 genome (Figure 4). This result was consistent with those of our previous studies, in which olive polyphenol-rich extract and herbal plant-derived pyrogallol-enriched fraction disrupted the SARS-CoV-2 genome [27,32]. The green tea catechins generated reactive oxygen species and damaged the DNA [35,36]. Such reactive oxygen species-dependent nucleotide damage is a possible mechanism of action of viral genome destruction. Ikigai et al. [37] demonstrated that EGCG damages phosphatidylcholine liposomes, and such a finding might indicate that catechins also impacted the virus envelope. In the present study, we could not perform electron microscopic observation of SARS-CoV-2 particles because of issues of safety in handling pathogens. However, such a structural observation of viral particles will provide information about the influence of TY-1 on the virus envelope.

Multiple previous studies evaluating the virucidal activity of TFs and green tea catechins have suggested that the number of galloyl and hydroxyl groups contributed to their virucidal activity, and TF3 and EGCG tended to show more potent antiviral activities than other catechins [15,18,19,27]. Therefore, further research to increase the content of TF3 and EGCG in TY-1 may contribute to the further enhancement of its virucidal activity.

## 4. Materials and Methods

### 4.1. Viruses and Cells

SARS-CoV-2 strains (2019-nCoV/Japan/TY/WK-521/2020 [ancestral strain], hCoV-19/Japan/QHN001/2020 [B.1.1.7 lineage; Alpha strain, GISAID ID: EPI_ISL_804007], hCoV-19/Japan/TY8-612-P1/2021 [B.1.351 lineage; Beta strain, GISAID ID: EPI_ISL_1123289], hCoV-19/Japan/TY7-501/2021 [P.1 lineage; Gamma strain, GISAID ID: EPI_ISL_833366], hCoV-19/Japan/TY11-927-P1/2021 [B.1.617.2 lineage; Delta strain, GISAID ID: EPI_ISL_2158617], and hCoV-19/Japan/TY11-330-P1/2021 [B.1.617.1 lineage; Kappa strain, GISAID ID: EPI_ISL_2158613]) were provided by the National Institute of Infectious Diseases (Tokyo, Japan). VeroE6/TMPRSS2 cells [38] established by the National Institute of Infectious Diseases were purchased from the Japanese Collection of Research Bioresources (No. JCRB1819, Osaka, Japan). SARS-CoV-2-inoculated VeroE6/TMPRSS2 cells were cultured in a previously described VGM [32].

### 4.2. Sample Preparation

TY-1 powder was provided by Yokoyama Food Co., Ltd. (Sapporo, Japan); its components are shown in Table S1. To prepare a 10 mg/mL TY-1 solution, 1.0 g TY-1 powder was dissolved in 100 mL phosphate-buffered saline (PBS), and the water-soluble layer was collected and stored at −80 °C. When the experiments were performed, to prepare TY-1 solutions with multiple concentrations, TY-1 solution was additionally diluted with PBS. As TY-1 powder contains 50% dextrin, 5 mg/mL dextrin solution was prepared as a solvent control by dissolving 0.5 g of dextrin in 100 mL PBS.

### 4.3. Evaluation of the Virucidal Activity of TY-1 against SARS-CoV-2

SARS-CoV-2 solution (~7.0 log_10_ TCID_50_/mL) was mixed with an equal volume of dextrin or TY-1. The final concentrations of dextrin and TY-1 in the mixture were 2.5 and 0.3–5.0 mg/mL, respectively. The mixtures were incubated at 22–25 °C from 1 min to 24 h and were then inoculated into cells; a 10-fold serial dilution of the cell culture medium, VGM, was performed. After incubation for 3 d, the cytopathic effect induced by SARS-CoV-2 infection was observed, and the viral titer (log_10_ TCID_50_/mL) was calculated using the Behrens–Kärber method [39]. The virucidal activities of TY-1 solutions of different concentrations were evaluated by comparing the difference in the viral titers between the dextrin group and each concentration of TY-1. The detection limits of the viral titers in the dextrin and TY-1 groups were determined based on the cytotoxic concentrations of those test solutions in virus-free conditions (Table S2). In Table S2, no cytotoxicity was defined as ≥ 80% cell survival, because abnormalities of cell morphology were not observed, and susceptibility to virus infection was maintained in these cells. The detection limits of viral titers in the groups treated with 2.5 mg/mL dextrin and 0.3–1.3 mg/mL TY-1 solutions were set to 1.25 log_10_ TCID_50_/mL, according to our viral titer calculation. The detection limits in the groups treated with 2.5 and 5.0 mg/mL TY-1 were set to 2.25 log_10_ TCID_50_/mL.

### 4.4. Evaluation of the Inhibitory Effect of TY-1 on SARS-CoV-2 Infection of Cells

SARS-CoV-2 solution was added into cells, and a twofold serial dilution was performed in the cell culture medium, VGM. At the same time, dextrin or TY-1 solution was added to the cell culture media. The final concentrations of dextrin and TY-1 in the cell culture media were 25 and 50 μg/mL, respectively; these concentrations did not show any cytotoxicity under virus-free conditions (Table S3). The cells to which virus was added were incubated at 37 °C for 1 h in the presence of dextrin or TY-1; then, the cell culture medium was removed. After washing with VGM, new VGM containing 1.55% carboxymethyl cellulose sodium salt (Nacalai tesque Co. Ltd., Kyoto, Japan) without dextrin or TY-1 was added, and the cells were further incubated at 37 °C for 3 d. The plaques were then observed, and the viral titer (× 10^5^ PFU/mL) was calculated.

### 4.5. Evaluation of the Impact of TY-1 on SARS-CoV-2 Structural Proteins

Western blotting targeting the S1 subunit of the S protein, the S2 subunit of the S protein, or the N protein was performed as previously described [32]. Briefly, SARS-CoV-2 solution (~7.0 log_10_ TCID_50_/mL), or each recombinant viral protein, was mixed with an equal volume of dextrin or TY-1. The final concentrations of dextrin and TY-1 in the mixture were 2.5 and 5.0 mg/mL, respectively. The final concentration of recombinant proteins in the mixture was 10 μg/mL. These mixtures were combined with sodium dodecyl sulfate (SDS) buffer with 2-mercaptoethanol (FUJIFILM Wako Pure Chemical Co., Ltd., Osaka, Japan) either immediately (0-h reaction time) or were incubated at 25 °C for 24 h prior to adding the SDS buffer (24-h reaction time). These samples were subjected to SDS–polyacrylamide gel electrophoresis using Mini-PROTEAN^®^ 3 Cell (Bio-Rad Laboratories Inc., Hercules, CA) and Power PAC^TM^ HC power supply (Bio-Rad Laboratories Inc.), and western blotting using Power PAC^TM^ HC power supply and Luminescent Image Analyzer LAS-3000 (FUJIFILM Co. Ltd., Tokyo, Japan) to detect the S1 subunit of the S protein, the S2 subunit of the S protein, and the N protein.

### 4.6. Evaluation of the Impact of TY-1 on the SARS-CoV-2 Genome

Real-time RT-PCR analysis targeting the SARS-CoV-2 N gene was performed as previously described [32]. Briefly, SARS-CoV-2 solution (~7.0 log_10_ TCID_50_/mL) was mixed with an equal volume of dextrin or TY-1. The final concentrations of dextrin and TY-1 in the mixture were 2.5 and 5.0 mg/mL, respectively. These mixtures were combined with ISOGEN-LS (Nippon Gene Co., Ltd., Tokyo, Japan) immediately (0-h reaction time) or were incubated at 25 °C for 24 h prior to adding ISOGEN-LS (24-h reaction time). These samples were subjected to real-time RT-PCR using LifeECO (Bioer Technology Co. Ltd., Hangzhou, China) and LightCycler^®^ 96 (F. Hoffmann-La Roche, Ltd., Basel, Switzerland). The primers and probe used in this study were NIID_2019-nCoV_N_F2, NIID_2019-nCoV_N_R2, and NIID_2019-nCoV_N_P2 [40].

### 4.7. Statistical Analysis

Student’s *t*-tests were performed to determine statistically significant differences between the dextrin and each of the TY-1 groups. *p* values of less than 0.05 were considered to be statistically significant.

## 5. Conclusions

In the present study, we showed that the tea leaf extract TY-1, which contains abundant tea polyphenols, including virucidal TFs and other green tea catechins, had time- and concentration-dependent SARS-CoV-2 inactivation activity. TY-1 induced the structural changes or destruction of the S protein S2 subunit and viral genome and inhibited the SARS-CoV-2 infection of cells. Since oral mucosal epithelial cells are one of the target cells of SARS-CoV-2 infection [41], TY-1 can be applied not only as an antiviral supplement but also as a troche, mouth rinse, or gargle. There may also be other ways of using TY-1 to prevent SARS-CoV-2 transmission. A previous report showed that green tea extract-doped masks blocked the passage of infective influenza A virus [42]. However, only the in vitro virucidal activity of TY-1 was tested in the current study. Further animal experiments and clinical tests in humans are needed to evaluate the actual use of TY-1. Nevertheless, our findings provide basic insights into the possible value of TY-1 as a highly safe antiviral agent, which may help reinforce the current SARS-CoV-2 control measures.

## Data Availability

Not applicable.

## Supplementary Materials

Table S1: Composition of 100,000 mg of TY-1 powder, Table S2: Cytotoxicity of dextrin and TY-1 (cell culture time in the presence of dextrin or TY-1: 3 d), Table S3: Cytotoxicity of dextrin and TY-1 (cell culture time in the presence of dextrin or TY-1: 1 h).

## Sample Availability

Samples of the compounds are available from the authors.
