# Supplementary material for: Severe Acute Respiratory Syndrome Coronavirus-2 Inactivation Activity of the Polyphenol-Rich Tea Leaf Extract with Concentrated Theaflavins and Other Virucidal Catechins
Source: Molecules. 2021 Aug 8;26(16):4803. doi: 10.3390/molecules26164803 (PMC8402090; doi:10.3390/molecules26164803)
Supplement: Supplementary file 1 [file molecules-26-04803-s001.zip › molecules-1285866-supplementary.pdf]

Supplementary information

Severe Acute Respiratory Syndrome Coronavirus-2 Inactivation Activity of The Polyphenol-Rich Tea Leaf Extract With Concentrated Theaflavins And Other Virucidal Catechins

Yohei Takeda [1,2], Kyohei Tamura [2], Dulamjav Jamsransuren [2], Sachiko Matsuda [2] and Haruko Ogawa [2,*]

[1] Research Center for Global Agromedicine, Obihiro University of Agriculture and Veterinary Medicine, 2-11 Inada, Obihiro, Hokkaido, 080-8555, Japan
[2] Department of Veterinary Medicine, Obihiro University of Agriculture and Veterinary Medicine, 2-11 Inada, Obihiro, Hokkaido, 080-8555, Japan
* Correspondence: author: hogawa@obihiro.ac.jp

Table S1. Composition of 100000 mg of TY-1 powder.

**Component**			Content
**Total polyphenols (~16000 mg)**	**Total theaflavins (1653 mg)**	Theaflavin (TF1)	1116 mg
		Theaflavin-3-gallate (TF2A)	294 mg
		Theaflavin-3′-gallate (TF2B)	140 mg
		Theaflavin-3,3′-digallate (TF3)	103 mg
	Total catechins (670 mg)	Epicatechin (EC)	350 mg
		Epicatechin gallate (ECG)	20 mg
		Epigallocatechin (EGC)	50 mg
		Epigallocatechin gallate (EGCG)	250 mg
	Gallic acid		1040 mg
	Other polyphenols		~12637 mg
Caffeine			1800 mg
Theanine			1300 mg
Dietary fiber			4400 mg
Dextrin			50000 mg

The chemical structures of theaflavins and catechins are shown in the article by Liu *et al.* [18].

Reference

18. Liu, S.; Lu, H.; Zhao, Q.; He, Y.; Niu, J.; Debnath, A.K.; Wu, S.; Jiang, S. Theaflavin derivatives in black tea and catechin derivatives in green tea inhibit HIV-1 entry by targeting gp41. *Biochim. Biophys. Acta Gen. Subj.* **2005**, *1723*, 270–281, doi:10.1016/j.bbagen.2005.02.012.

Supplementary Materials and Methods

Evaluation of Cytotoxicity of Dextrin and TY-1

The cytotoxicity of dextrin and TY-1 to VeroE6/TMPRSS2 cells were evaluated using the following two different cell culture procedures.

(1) Dextrin or TY-1 of different concentrations was added to the cell culture medium, VGM. VeroE6/TMPRSS2 cells were cultured in this cell culture medium in 96-well plates (Nalge Nunc International Co., NY, USA) at 37℃ for 3 d. The number of live cells was evaluated based on the amount of ATP present in the cells, which was measured using Cell Titer-Glo® Luminescent Cell Viability Assay (Promega Co., WI, USA) and the Glomax® -Multi+ Detection System (Promega Co). The cell viability (%) was calculated using the following formula: [luminescence in the test solution-added well] / [luminescence in the test solution-free well] × 100. Based on the result obtained, the detection limit of the viral titer in the experiment shown in Figure 1 was determined.

(2) Dextrin or TY-1 of different concentrations was added to the cell culture medium, VGM. VeroE6/TMPRSS2 cells were cultured in this cell culture medium in 96-well plates at 37°C for 1 h, after which the cell culture medium was removed. After washing with VGM, new VGM without dextrin or TY-1 was added, and the cells were further incubated at 37℃ for 3 d. Then the cell viability was calculated as described above. Based on the results obtained, the concentrations of dextrin and TY-1 used in the experiment shown in Figure 2 were determined.

Table S2. Cytotoxicity of dextrin and TY-1.

(Cell culture time in the presence of dextrin or TY-1: 3 d).

**Conc.** of dextrin (g/ml).	**0.0**	**31.3**	**62.5**	**125.0**	**250.0**	**500.0**
**Cell survival (%)**	100.0	98.1	89.9	99.0	101.7	103.9

**Conc. of TY-1 (g/mL)**	**0.0**	**31.3**	**62.5**	**125.0**	**250.0**	**500.0**	**1000.0**
**Cell survival (%)**	100.0	82.6	83.6	85.8	72.8	30.5	1.1

Table S3. Cytotoxicity of dextrin and TY-1.

(Cell culture time in the presence of dextrin or TY-1: 1 h)

**Conc. of dextrin (g/mL)**	**0.0**	**31.3**	**62.5**	**125.0**
**Cell survival (%)**	100.0	103.6	99.2	97.4

**Conc. of TY-1 (g/mL)**	**0.0**	**50.0**	**100.0**	**200.0**	**400.0**	**800.0**
**Cell survival (%)**	100.0	100.2	91.5	72.9	38.1	6.5
